# Evaluation of Utilizing the Distinct Genes as Predictive Biomarkers in Late-Onset Alzheimer's Disease

**DOI:** 10.1055/s-0042-1743570

**Published:** 2022-03-08

**Authors:** Sercan Kenanoglu, Nefise Kandemir, Hilal Akalin, Nuriye Gokce, Mehmet F. Gol, Murat Gultekin, Emel Koseoglu, Meral Mirza, Munis Dundar

**Affiliations:** 1 Department of Medical Genetics, Faculty of Medicine, Erciyes University, Kayseri, Turkey; 2 Department of Medical Genetics, Diskapi Yildirim Beyazit Training and Research Hospital, Ankara, Turkey; 3 Department of Neurology, Faculty of Medicine, Erciyes University, Kayseri, Turkey

**Keywords:** *PARP1*, *POLB*, *HTRA2*, *SLC1A2*, *HS1BP3*, *DRD3*

## Abstract

Alzheimer's disease (AD) is a neurodegenerative disease that is characterized by a devastating decline in cognitive activities among all types of dementia, and it severely affects the quality of life. Late-onset AD (LOAD) occurs after the age of 65 years and develops sporadically. Although aging comes first along the main risk factors underlying LOAD, disease-causing susceptibility genes have been associated with disease pathogenesis. In our study, we included the genes *PARP1* , *POLB* , *HTRA2* , *SLC1A2* , *HS1BP3* , and *DRD3* to be investigated in LOAD patients based on their expression levels. Within this framework, we aimed to determine the possible functions of these genes in the pathophysiology of the disease. We investigated whether the utilization of these genes as biomarkers in the early diagnosis of LOAD may help the treatment scheme to be applied in the clinic. We involved 50 individuals in the study and collected peripheral blood samples from the patients and control groups for molecular genetic analysis. Subsequently, RNA was extracted from the peripheral blood samples, and expression analyzes were performed using qualitative reverse transcription polymerase chain reaction. The results obtained were evaluated by using proper statistical methods. Our results demonstrated that there was no difference between patient and control groups in terms of *HTRA2* , *DRD3* , *HS1BP3* , and *POLB* genes. The expression levels of the *SLC1A2* and *PARP1* genes were significantly lower in the patient group compared with the control group. In conclusion, we presume that the *PARP1* and *SLC1A2* genes can be utilized as molecular biomarkers for LOAD.

## Introduction

Alzheimer's disease (AD) is an autosomal dominant genetic disorder corresponding to 60 to 70% of all types of dementia. 1 The disease is characterized by a devastating decline in cognitive activities and affects approximately 40 to 50 million people worldwide, leaving patients in need of care as the disease progresses. In the light of current data, it is estimated that this incidence will increase thrice by 2050. 2

There are two main types of AD which have a very high heterogeneity and complexity: early-onset AD (EOAD) and late-onset AD (LOAD). While the type of AD that occurs before the age of 65 years is considered as EOAD, LOAD, also known as senile AD, is a form that arises after 65 years of life and evolves sporadically. Although the heritability is relatively low, the effect of aging, susceptibility genes, and environmental factors are considered among the most prominent risk factors underlying LOAD. 3 4 The Genome-Wide Association Studies (GWAS) have also been an important guide for LOAD in the identification of these risk factors, and more than 20 genetic loci have been identified with these studies, so far. Although its pathophysiology is based on broad molecular bases, apolipoproteins and genes that share common functions and pathways associated with lipid homeostasis and endocytosis are major targets associated with this form of the disease. 5 6 In addition, today's biotechnological developments provide new opportunities for the investigation of many neurodegenerative disorders, including AD, and contribute to the development of targeted therapies, as well as producing preventive strategies by drawing attention to the importance of early diagnosis. 7

*PARP1* (poly[ADP-ribose] polymerase 1) is a 47 kb protein-coding gene located on chromosome 1 (1q42.12). The protein encoded by this gene is poly ADP-ribosyl transferase, a chromatin-associated enzyme which is responsible for the modification of various nuclear proteins by poly (ADP-ribosyl)ation. PARP1 is activated in response to DNA damage and functions as a DNA damage sensor in physiological processes such as DNA repair, genomic stability, cell survival, and apoptosis; therefore, it has been associated with neurodegenerative diseases including AD which is characterized by the death of neuronal cells. 8 9

*POLB* (DNA polymerase β) gene is mapped to chromosome 8p11.21, and the protein encoded by this gene is an important type of DNA polymerase involved in base excision repair, also defined as gap-filling DNA synthesis. The encoded protein is normally found in the cytoplasm and acts as a monomer; however, it is translocated to the nucleus in case of DNA damage. 10 Since POLB is also involved in meiosis and repair of double-strand breaks, this enzyme has also been associated with synapse and recombination-related meiotic events.

*HTRA2* (high-temperature requirement protein A2), a gene localized on the short arm of chromosome 2 (2p13.1), encodes a serine protease which influences the development of apoptotic processes and various neurodegenerative diseases. 11 This protein, a member of the high-temperature requirement serine protease A, which acts as a protective chaperon while in the mitochondria, becomes a proapoptotic molecule as soon as it releases into the cytosol and allows apoptosis to occur in both caspase-dependent and caspase-independent manners. 12

*SLC1A2* (solute carrier family 1 member 2) gene, also known as EAAT2 (excitotoxic amino acid transporter 2) is localized on chromosome 11p13 and has an essential function in the regulation of extracellular glutamate levels in the central nervous system. 13 This regulation is achieved by clearing several neurotransmitters involved in neural destruction, including glutamate, from the extracellular space. It has been observed that the defects occurring in this regulation mechanism lead to the cell moving toward neuronal death.

*HS1BP3* (hematopoietic-specific protein 1 binding protein 3) gene is localized on 2p24.1 and was first identified as tyrosine 3-monooxygenase/tryptophan 5-monooxygenase activation protein, a protein transporter. 14 Early findings have shown that *HS1BP3* has a role in interleukin-2 signaling and gene expression in many tissues including the brain. In recent studies, it has been clearly shown that the autophagy process is dysregulated in AD. 15 16

*DRD3* (dopamine receptor D3) is a gene located on the long arm of chromosome 3 (3q13.31) which encodes the D3 subtype of dopamine receptors. *DRD3* is found in the limbic cortex and takes a priority function in the regulation of cognitive and behavioral regulation. Therefore, the alterations in the *DRD3* gene have been linked with diversified mental disorders and dementia symptoms of AD. 17

In our study, we aimed to investigate the mRNA expression levels of the genes including *PARP1* , *POLB* , *HTRA2* , *SLC1A2* , *HS1BP3* , and *DRD3* in LOAD patients and to identify their possible functions in the pathophysiology of the disease. Considering the available data in the literature on these genes, it is obviously seen that they have strong effects in various neurodegenerative diseases, including AD. Therefore, we hypothesized that early detection in LOAD might help slow down the progression and reduce the symptoms of the disease, also helping to utilize these genes as a biomarker in blood in guiding the treatment to be applied. In this way, it is predicted that the economic burden of the treatment may decrease, and the life quality of the patients can increase.

## Materials and Methods

### Patients and Controls

The individuals included in the study applied to the Department of Neurology, and after the neurological examinations were made, they were referred to the Department of Medical Genetics. Patients diagnosed with AD by mini–mental state examination (MMSE) test and other neurological ancillary tests were included in the study. 18

A total of 50 individuals were included in the study, 22 patients (44.00%) and 28 as control groups (56.00%). The patient group consisted of 17 males (77.27%) and 5 females (22.73%), while the control group consists of 15 males (53.57%) and 13 females (46.43%). All individuals were included in the analysis and the study was completed. The age interval of the patient group was between 65 and 83 years and the mean age was 72.18 years. The control group consisted of individuals whose ages were between 65 and 78 years and the mean age was 68.86 years with no dementia, as well as any psychiatric, neurological, and other internal oxidative stress. The distribution of demographic characteristics within both groups were shown in Table 1 .

**Table 1:** Distribution of demographic characteristics within the patient and control groups

		Patient group ( *n* = 22)	Control group ( *n* = 28)
Gender ( *n* )	Male	17	15
	Female	5	13
Age (y) Mean ± standard deviation		71.95 ± 5.55	69.6 ± 4.06

Informed consent was obtained from all participants. Consent forms were signed by patients/legal guardians who gave permission to use the patients' clinical history information and genetic test results as part of an education process and as material for publication in the scientific journals. The study was performed with all the required ethical approval permissions which were obtained from the Erciyes University Ethical Committee of Clinical Studies (date and number of approval: February 2, 2019/81).

### Sample Collection and RNA Extraction

Peripheral blood samples (9–10 cc) were collected from the patients and control groups in Ethylenediaminetetraacetic acid (EDTA)-containing tubes for molecular genetic analysis. The test was performed by the following steps: leukocyte isolation according to TRIzol Reagent method, total RNA extraction, cDNA synthesis, quantitative real-time polymerase chain reaction (qRT-PCR), followed by the statistical analysis and interpretation of the data.

Leukocytes were isolated from 9 to 10 cc peripheral blood samples obtained from patients using commercially available TRIzol reagent (TriPure Isolation Reagent, Roche). Afterward, total RNA was extracted by using the chloroform–phenol extraction method according to the manufacturers' instructions. 19

### Quantitative Real-Time Polymerase Chain Reaction and the Expression Analysis

Following the RNA extraction, cDNA synthesis was performed using the Transcriptor High Fidelity cDNA Synthesis Kit (Roche Diagnostics, GmbH, Mannheim, Germany). For the reaction, random hexamer primers were used.

The qRT-PCR was performed on LightCycler 480 II (Roche Diagnostics Ltd. Rotkreuz, Switzerland) and the analyses were performed with LightCycler 480 Software (release 1.5.0 SP4) based on the 2 ^-ΔΔCt^ formula. Each sample was performed as duplicated, two negative controls and calibrators were used in each run. The Real-Time ready Catalog Assays (Roche Diagnostics, GmbH, Mannheim, Germany) and Real-Time ready Designer Assays (Roche Diagnostics, GmbH, Mannheim, Germany) were used. All data were normalized to the reference gene, human β-actin ( *ACTB)* .

### Statistical Analysis

The data were analyzed using IBM SPSS Statistics 22 software (IBM SPSS Statistics for Windows, Version 22.0. Armonk, New York, New York, United States: IBM Corp. Released 2013 software). Graphs were drawn with GraphPad Prism 8.0 (GraphPad Software, California, United States) software. In evaluating the suitability of the data for normal distribution, histogram, quantile-quantile plot, and Shapiro–Wilk tests were used. The nonparametric Mann–Whitney *U* -test was used when the normal distribution was not observed, since the data did not show normal distribution. A Chi-square test was used to compare the distribution according to the gender of the participants. Spearman's correlation test was used to investigate the relationship between age and expression levels between groups. The number of units ( *n* ), percentage (%), mean, and standard deviation values were given as summary statistics. According to the test results, *p* < 0.05 considered to be statistically significant.

## Results

**Fig. 1:**
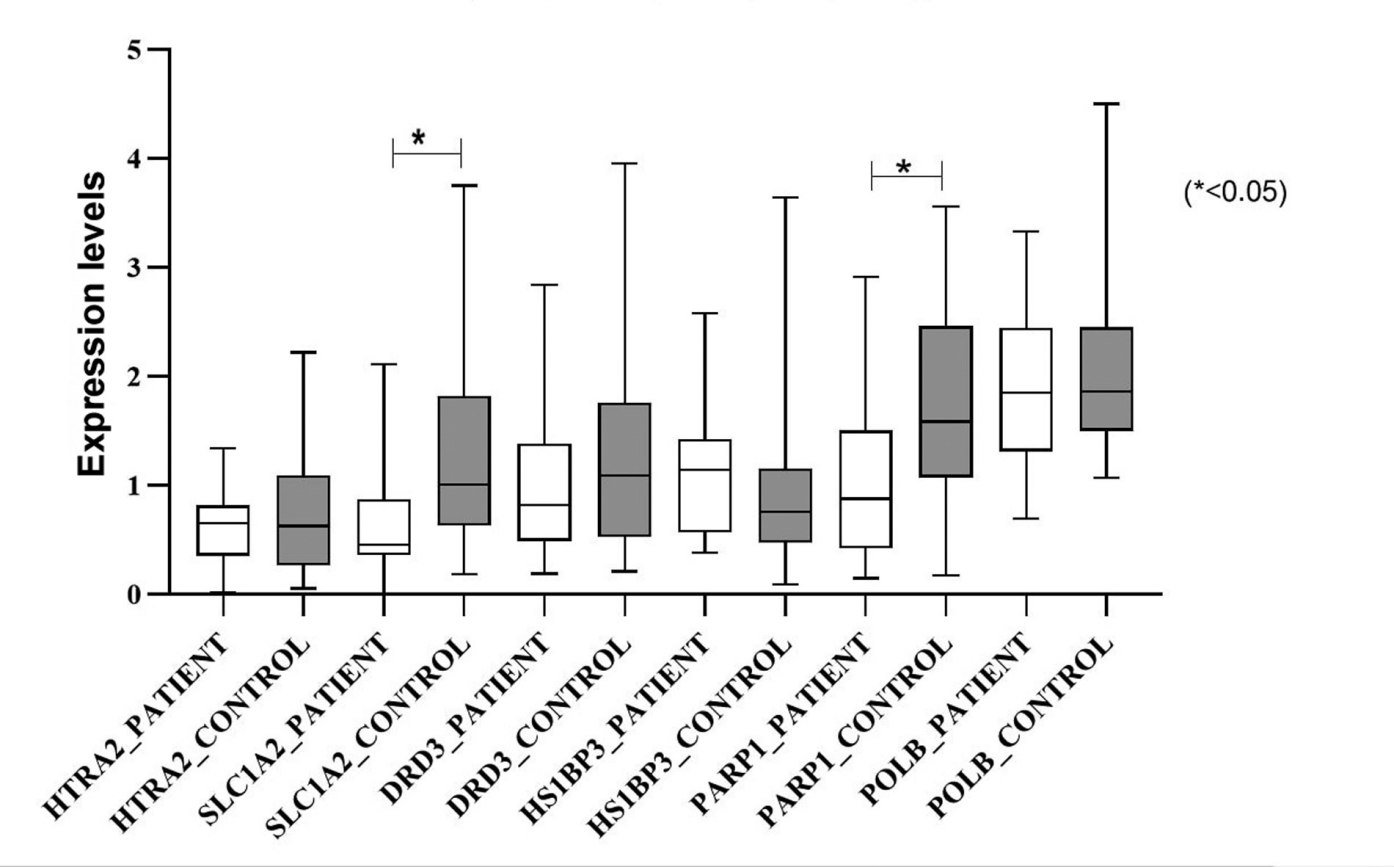
Expression levels of all genes included in the study in the patient and control groups.

A total of 50 individuals participated in the study and were classified as AD patient group ( *n* = 22) and healthy control group ( *n* = 28). The patient group that made up our cohort consisted of 17 males and 5 females, while the control group consisted of 15 males and 13 females. While the mean age of the patient group was 72.18 years, the mean age of the control group was calculated as 68.86 years, and there was no significant difference in age and gender between these two groups. No significant difference was found between the LOAD stages and the expression levels of genes. In terms of gene expression levels, there was no difference between the patient and control groups in *HTRA2* , *DRD3* , *HS1BP3* , and *POLB* genes. However, the expression levels of the *SLC1A2* and *PARP1* genes were significantly lower in the patient group compared with the control group ( *p* < 0.05), respectively ( *p* = 0.048 and 0.044). The expression levels of all genes included in the study in the patient and control groups are shown in Fig. 1 . Gene expression levels of both groups were given in Table 2 . Interestingly, a significant positive correlation was found between *SLC1A2* and *HTRA2* ( *p* < 0.01), *DRD3* ( *p* < 0.01), and *HS1BP3* ( *p* < 0.05) in our control group. However, this correlation was not observed in our patient group.

**Table 2:** Gene expression levels of study groups

	*HTRA2*	*SLC1A2*	*DRD3*	*HS1BP3*	*PARP1*	*POLB*
Control group	2.26 ± 8,21	1.83 ± 2.09	1.46 ± 1.93	2.19 ± 3.35	1.69 ± 0.89	2.19 ± 1.08
Patient group	0.79 ± 0,85	1.01 ± 1.20	1.09 ± 0.81	1.07 ± 0.57	1.11 ± 0.79	2.08 ± 1.02
*p* -Value	0.89	0.048	0.95	0.53	0.044	0.821

In the patient group, there is a positive correlation between *PARP1* and *SLC1A2* ( *p* < 0.05). However, there is a negative correlation between *POLB* and *HTRA2* ( *p* < 0.05) as well. Similarly, there is a negative correlation between *PARP1* and *HS1BP3* ( *p* < 0.05).

**Fig. 2:**
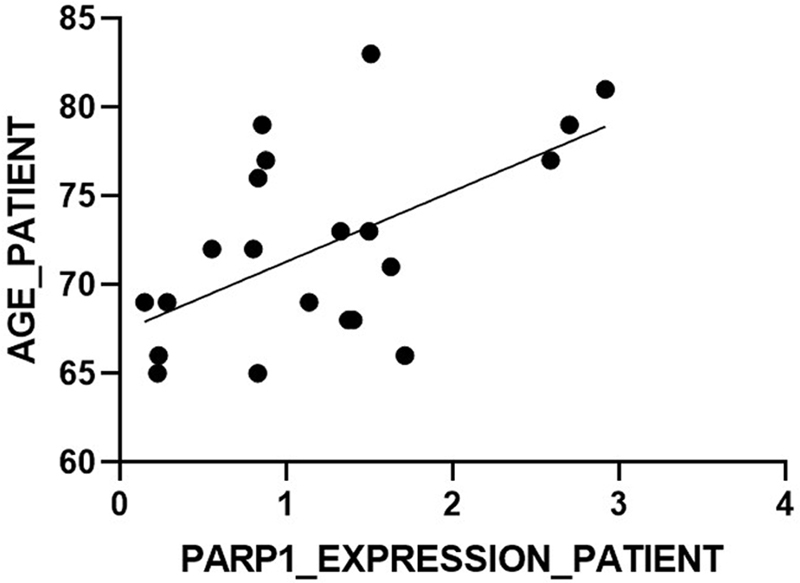
*PARP1* gene expression level and age correlation graph in the patient group ( *p* < 0.05).

For the control group, a positive correlation was found between *DRD3* and *HTRA2* ( *p* < 0.05). Additionally, a positive correlation was found between the *PARP1* gene and age ( *p* < 0.05; Fig. 2 ). Correlation analyzes of gene expression values within themselves and with sample ages were performed. Significant *p* -values and correlation coefficient (rho) values are shown in Table 3 .

**Table 3:** The *p* - and rho values of correlation analysis results

	Control group				Patient group			
Parameters	*SLC1A2* and *HTRA2*	*SLC1A2* and *DRD3*	*SLC1A2* and *HS1BP3*	*DRD3* and *HTRA2*	*PARP1* and *SLC1A2*	*PARP1* and *HS1BP3*	*POLB* and *HTRA2*	*PARP1* and age
*p* -Value	0.000284	0.000202	0.013	0.008	0.025	0.026	0.045	0.026
Correlation coefficient (rho)	0.645	0.677	0.471	0.511	0.542	−0.539	−0.493	0.539

## Discussion

In our study, we aimed to determine the possible differences in the expression levels of *PARP1* , *POLB* , *HTRA2* , *SLC1A2* , *HS1BP3* , and *DRD3* genes which are stated in the literature to be associated with neurodegeneration, and to investigate whether they can be biomarkers in the clinical evaluation of LOAD patients. The study group we have formed consisted of two groups, the patient and control group, both of which contain individuals who are over 65 years of age. Our understanding that these genes play an important role in the development of neurodegenerative diseases has suggested that some differences may arise in the molecular pathways of AD.

LOAD is a progressive neurodegenerative disorder that negatively affects the cognitive functions of many elderly people around the world and it ranks first among dementia-related deaths. 20 Several symptomatic medications are currently available including acetylcholinesterase inhibitors that partially alter the effects of the disease on cognition; however, their efficacy in mild cognitive impairment and prodromal AD is still controversial. 21

In studies, the prevalence of LOAD is 0.6% in people aged 65 to 69 years. It has also been reported that this prevalence is 22.2% in people aged 90 years and over. It is estimated that this ratio will increase further with the increase in the average life expectancy. Although genetics is not the primary factor in the development of LOAD, GWAS have led researchers in this field to identify novel risk genes to elucidate the molecular mechanism of this disease. 22 GWAS studies have enabled us to understand that AD has a very strong genetic and epigenetic component and made it possible to evaluate the detected variants in a wider spectrum. It is thought that studies in different populations will underline the importance of new genetic risk factors of AD and pave the way for in-depth approaches for mapping functional variants in distinct genes. 23

PARP1 constitutes two zinc finger models by incorporating the DNA-binding domain (DBD) which mediates high-affinity binding to single or double chain breaks in DNA. After binding to the damaged DNA, PARP1 binds covalently to the nuclear receptor protein by forming homodimers and catalyzes the cleavage of NAD ^+^ to nicotinamide and ADP-ribose to synthesize long-branching poly (ADP-ribose) polymers. 24 25 Studies on mice have shown that NAD ^+^ is regulated by various protein–protein interactions during the aging process, and it has been observed that DNA damage accumulates with the binding of PARP1 inhibitors on PARP1 with the decrease in NAD ^+^ concentration in aged mice. 26 It has been previously known in the literature that PARP1, which is a marker of DNA damage, causes neurodegeneration due to its effect on amyloid plaque formation in the brains of AD patients. 27 Based on these findings, it is thought that the decrease of PARP1 with age causes the DNA repair rate to decrease and may affect the progression of the disease and even the response to treatment. Our results showed that the expression level of the *PARP1* gene in LOAD patients was lower than in the control group. This might be due to the fact that PARP1 has a critical role in DNA repair, and the effect of the loss of function in this gene results in the development of LOAD.

Another crucial DNA repair gene that we predict may have a role in AD is *POLB* . Based on the available data in the literature, it can be implied that the accumulation of oxidative DNA lesions and defects in DNA repair make an important contribution to the progression of LOAD. 28 29 30 31 POLB is one of the most critical enzymes involved in the base excision repair (BER) pathway which is a substantial repair pathway against DNA lesions and reactive oxygen species (ROS). Lower BER activities were found in the nuclear and mitochondrial lysates of the cortex and cerebellum regions of the brains of AD patients. It has been found that this low level of BER makes neurons more susceptible to cell death and induces Neurofibrillary tangles (NFT) formation with amyloid plaques. 30 32 33 34 On the other hand, it has been reported that the presence of other early affected brain regions in the pathophysiology of the disease should also be confirmed with additional studies. 31 The role of the POLB in the pathogenesis of AD has also been demonstrated in produced transgenic animal models. Neurodegeneration was observed in a DNA Polβ ^+/−^ mouse model as a result of the suppression of the DNA polymerase enzyme. Based on the findings, dysregulations in DNA Polβ have been linked with the characteristic features of AD such as neuronal dysfunction, cell death, impaired memory, and synaptic plasticity. 35 Other studies have shown that POLB depletion in 3xTg AD/Polβ ^+/−^ mice not only causes memory impairment but also exacerbates neurodegeneration and AD phenotypes, including hippocampal synaptic plasticity and olfactory perception. 36 37 38 39 In our study, no significant difference was found between the patient and control groups in the *POLB* gene expression level in peripheral blood. This may be because *POLB* expression levels were unaffected in the blood or our sample was not large enough. In addition, considering the animal experiments in the literature and studies conducted on human brain tissue, our results suggest that this gene might cause a significant change only in brain tissue. Furthermore, taking into account the differences in protein level, it is also necessary to follow-up on how much of the expressed genes are converted into protein. For all these reasons outlined, it is thought that additional studies to be performed will provide more precise results in ensuring this follow-up.

HTRA2, a member of high-temperature requirement serine protease A family, has also been associated with neurodegenerative diseases. In the presence of an apoptotic stimulus, HTRA2 is released from mitochondria to cytosol and interacts with the cytosolic inhibitor of apoptotic proteins (IAPs) and hinders its caspase inhibition. 40 41 At the cellular level, the proapoptotic function of this protein is at the forefront, and additionally, gene deletion studies have shown that the disruption of the functionality of HTRA2 leads to several neurodegeneration phenotypes. Although the mechanism underlying cell death, a cause of neurodegenerative diseases has not been clarified yet, studies revealed that the active form of HTRA2 is significantly increased in the brains of AD patients. 42 Westerlund et al associated neuronal damage between the disease and OMI/HTRA2 protease activity. In addition, the level of the processed form of OMI/HTRA2 (hypothetically active form) in the frontal cortex of AD patients was found to be lower than controls. Surprisingly, functional analysis showed that despite the general decrease in OMI/HTRA2 protein levels, there was a significant increase in OMI/HTRA2 protease activity in the frontal cortex of the brain when compared with patients and controls. 43 When studies on transgenic animal models are examined, it was observed that mice with mutant or knocked-out HtrA2/Omi develop neurodegeneration owing to mitochondrial destruction. This clearly shows that a functional HtrA2/Omi is needed to prohibit neurodegeneration resulting from mitochondrial destruction. 11 44 Considering the fact that OMI/HTRA2 is found to be increased in the brains of AD patients, our findings clearly show that no significant difference was observed between HTRA2 expression levels in the patient and control groups. We can propose that this is because we compared expression levels only on blood material. In the current studies, an expression study was conducted directly from postmortem brain tissues of AD patients. Therefore, the expression level of the *HTRA2* gene in the blood is not informative in terms of prognostic or diagnostic in LOAD.

*SLC1A2* gene is known for an important role in the regulation of extracellular glutamate levels. Madeira et al demonstrated that impairment in glutamate-mediated excitatory signaling leads to several impairments in the brain function of Alzheimer's patients. 45 When the pathogenesis of AD was examined, it was observed that the postsynaptic glutamate receptors were overstimulated as a result of glutamate not being able to clear the synaptic cleft, thereby promoting neuronal death. Additionally, it was observed that alternative splicing in the *SLC1A2* gene in the disease phenotype was reflected in its pathological effect. 46 Considering the animal studies conducted, as a result of the *SLC1A2* null mutation, increased glutamate levels in the forebrain regions such as the hippocampus have shown fatal spontaneous seizures and acute cortical damage associated with overactivation of N-methyl-D-aspartate (NMDA) and glutamate receptors. 47 In our patient group, *SLC1A2* levels were found to be significantly lower than the control group. Considering that this gene is responsible for the regulation of glutamate level, we suppose that the *SLC1A2* gene and glutamate levels may be a marker on LOAD and should be investigated within the framework of more comprehensive studies.

HS1BP3 is involved in the members of the tyrosine 3-monooxygenase/tryptophan 5-monooxygenase activation protein family and is mainly secreted in cerebellar purkinje cells and hippocampal pyramidal cells in the brain. Tyrosine and tryptophan hydroxylase, two important enzymes of the catecholamine and serotonin pathways, are responsible for Ca ^2+^ /calmodulin-dependent protein kinase activation. 14 Another important mechanism underlying AD is dysregulations in the autophagy process, and in one study, HS1BP3 was shown to negatively regulate autophagy. 15 Inaccuracies during autophagosome formation have been associated with neurodegenerative disorders including AD, and it has been observed that the survival of healthy cells is also affected as a result of this uncontrolled autophagy. 48 According to our results, a decrease is observed in the expression levels of the *HS1BP3* gene in the patient group compared with the control group. However, this was not found to be statistically significant. We believe that a more effective result can be obtained by increasing the number of our cohort.

*DRD3* gene, plays a role in cognitive and behavioral regulation. Montoya et al focused on the dynamics of microglia and astrocyte activation, considering the systemic inflammation of DRD3 signaling, and they emphasized that astrogliosis has an important place in the pathogenesis of neurodegenerative diseases such as Parkinson's disease (PD) and AD. 49 In addition, some researchers have reported that a functional change in the *DRD3* gene affects cognitive and psychiatric symptoms in AD through a linkage disequilibrium with other genetic variations in the promoter region of the relevant gene. And in this regard, they thought that it would play a role in the development of paranoid and delusional thinking which varies according to the severity of the disease. 17 50 Kandemir et al studied *DRD3* gene expression in essential tremor (ET), a neurodegenerative disease, and found it significantly lower in the patient group than in the control group. They stated that the low dopamine level in the ET group may be due to polymorphisms or mutations in this gene. 51 In our study, we investigated the mRNA expression level of *DRD3* in light of this information and found that there was no significant difference between the patient and control groups. Further studies are needed at the protein and tissue level by increasing the number of the study population.

## Limitations

The limitation of this study is that expression levels were only examined at the blood level and could not be examined in brain tissues. Additionally, it should also be noted that the sample size was not large enough because the number of patients who applied to the dementia policlinic at the time of the study was limited, therefore similar studies with a wider sample size are required to verify the results obtained. Likewise, the single-center nature of our study is among the other limitations of this study. Considering the conditions in our study, it was not possible to analyze the expression level of these genes in human brain tissue, thus it aimed to conduct further studies by comparatively examining both blood and brain tissue in the transgenic animal model. We believe that additional studies at the protein level should be planned to expand the studies at the gene expression level and to confirm the data we have obtained.

## Conclusion

Based on the findings, we obtained from this study and the data evaluation, we can presume that the *PARP1* and *SLC1A2* genes can be molecular biomarkers for LOAD. In addition, the pathways affected by these genes and glutamate levels affected by *SLC1A2* should be studied in detail, and further studies should be conducted to support its use as a molecular marker in the early diagnosis of AD. Significant correlation values may be due to the small number of patients, and these results may be misleading. We presume that studies should be strengthened by increasing the number of samples to verify these values. Further studies in this area are planned. Finally, it is also important to emphasize one point that protein levels of all genes included in the study will be studied as advanced research, and the results obtained will be supported by animal experiments.
